# PTEN restricts IL-21R signaling in GC B cells and suppresses their differentiation to plasma cells

**DOI:** 10.1093/jimmun/vkaf160

**Published:** 2025-08-07

**Authors:** Alexander R Maldeney, Wenxia Jiang, Laura Conter, Daniel J Wikenheiser, William F Hawse, Anandkumar Patel, Weixun Peng, Mark J Shlomchik, Wei Luo

**Affiliations:** Department of Microbiology and Immunology, Indiana University School of Medicine, Indianapolis, IN, United States; Department of Microbiology and Immunology, Indiana University School of Medicine, Indianapolis, IN, United States; Department of Immunology, University of Pittsburgh School of Medicine, Pittsburgh, PA, United States; Department of Immunology, University of Pittsburgh School of Medicine, Pittsburgh, PA, United States; Department of Immunology, University of Pittsburgh School of Medicine, Pittsburgh, PA, United States; Department of Microbiology and Immunology, Indiana University School of Medicine, Indianapolis, IN, United States; Department of Microbiology and Immunology, Indiana University School of Medicine, Indianapolis, IN, United States; Department of Immunology, University of Pittsburgh School of Medicine, Pittsburgh, PA, United States; Department of Microbiology and Immunology, Indiana University School of Medicine, Indianapolis, IN, United States

**Keywords:** germinal center B cell, plasma cell differentiation, PTEN, signaling

## Abstract

The germinal center (GC) reaction is essential for generating high-quality humoral memory. Positively selected GC B cells must decide whether to remain in the GC for further affinity maturation or differentiate into memory or plasma cells (PCs). Previously, we identified IL-21R and CD40 signaling as critical promoters of GC B cell effector differentiation. However, the mechanisms regulating the strength of these signals remain unclear. PTEN, a negative regulator of PI3K signaling, is markedly upregulated in GC B cells. To explore the role of PTEN in GC B cell fate decisions, we used a tamoxifen-driven Cre system to delete PTEN in B cells after GC reaction was fully established. We found that PTEN deletion in ongoing GC B cells led to significantly increased differentiation into PCs, while having limited effects on class switching or memory precursor differentiation. These results were further confirmed using a GC B cell transfer system, where wild-type and PTEN-deficient GC B cells were transferred into the same recipient mice. Mechanistically, PTEN deletion or inhibition in established GC B cells resulted in more sustained IL-21R signaling and enhanced CD40 signaling—both known to promote PC differentiation. Interestingly, Peyer’s patch GC B cells exhibited higher PTEN levels than de novo GC B cells generated through immunization. Notably, PTEN deficiency selectively expanded GC B cells in Peyer’s patches but had no impact on those induced by immunization. These findings reveal previously unrecognized roles for PTEN in regulating GC B cell signaling and limiting their differentiation into PCs.

## Teaser

The Editors have selected this article as a highlight of the issue.

## Introduction

Germinal center (GC) reaction generates long-lived humoral memory compartments that protect us from infectious pathogens. GC B cells constantly go through affinity-based and signaling-guided selection.^1^ The successfully selected of GC B cells may face three possible fate choices: staying in the GC for further selection, differentiation towards plasma cells (PCs) or differentiation towards memory B cells (MBCs). Despite recent advances in understanding GC selection, the signaling that controls fate decision of selected GC B cells is poorly understood. Addressing this knowledge gap is fundamental for developing approaches to manipulate GC response, which can be very useful for improving vaccine efficacy. Phosphatase and tensin homolog (PTEN) has major impacts on multiple biological functions through regulating inositol lipid signaling and PI3K-AKT-mTOR pathway.^2^ PTEN regulates early B cell development in the bone marrow,^3^ and is essential for maintaining B cell anergy to prevent autoimmunity.^4^ Viral infection can upregulate PTEN in B cells to suppress antibody responses.^5^  *Pten* deletion during early B cell development via CD19-Cre resulted in altered B cell maturation, B cell hyperactivation and severe blockade of GC formation.^6^^,^^7^ Deleting *Pten* in B cells during isotype class-switch recombination (using Cγ1-Cre) blocked switching to IgG, attributed to reduced expression of activation-induced cytidine deaminase (AID).^8^ However, since class switching and Cγ1 transcription can occur before GC initiation,^9^^,^^10^ the Cγ1-Cre allele would affect B cell-expressed PTEN prior to GC differentiation.

We previously discovered that PTEN protein (but not mRNA) is highly elevated in GC B cells, which could play a role in reprogramming B cell receptor (BCR) signaling in GC B cells to facilitate affinity-based selection.^11^^,^^12^ In addition to its role in regulating B cell responses in viral infection,^5^ PTEN is also dysregulated in subsets of GC-derived B cell lymphomas.^13^^,^^14^ However, the role PTEN signaling in the function and fate-decisions of established GC B cells remains undefined. Previous studies utilizing CD19-Cre or Cγ1-Cre systems were unable to address this question due to Cre-mediated PTEN deletion occurring in B cells prior to GC establishment. To overcome this limitation, we employed an inducible Cre-mediated conditional deletion model and a recently developed GC B cell modification and transfer system to investigate the role of PTEN in established GC B cells. These experiments identified a pivotal role for PTEN in limiting the differentiation of GC B cells into PCs. Mechanistically, we further showed in GC B cells that PTEN regulates both IL-21R and CD40 signaling, which in turn influences PC differentiation.

## Materials and methods

### Mice and immunization

*Pten* ^flox^ (B6.129S4-*Pten^tm1Hwu/J^* Strain no. 006440) mice were purchased from Jackson Laboratory, and were crossed to hCD20^TamCre^ mice on the B6 background.^11^ C57/B6 wild-type mice were obtained from the In Vivo Therapeutics Core of the Indiana University Simon Cancer Center. In total, 6- to 12-week-old mice were immunized s.c. with 100 μg 4-hydroxy-3-nitrophenylacetyl-ovalbumin (NP-OVA, LGC Biosearch Technologies) adjuvanted by AddaVax (InvivoGen, Cat: vac-adx-10) in 1:1 ratio. GC B cell transfer experiments were performed with B1-8i knock-in (“B1-8i”) BALB/c as previously described.^11^^,^^15^^,^^16^ 6- to 12-week-old B1-8i mice were immunized i.p. with 50 μg 4-hydroxy-3-nitrophenylacetyl-chicken gamma globulin (NP-CGG) precipitated in alum. Mice were analyzed at the indicated time points as described in figures. Mice were housed under specific-pathogen-free conditions and supervised by Institutional Animal Care and Use Committees of Indiana University and University of Pittsburgh.

### Tamoxifen-induced deletion of *Pten*

*Pten* ^fl/fl^ hCD20^TamCre^ mice and control *Pten^+/+^* hCD20^TamCre^ mice were gavaged with 0.5 mg Tamoxifen in 100 μl corn oil on day 7 post NP-OVA immunization and analyzed on day 14. The loss of PTEN protein was confirmed by flow cytometry analysis with a PTEN antibody (clone: D4.3, Cell Signaling Technology).

### PTEN knockdown in GC B cells

GC B cells were isolated from day 14 NP-CGG immunized B1-8i mice by negative selection using a biotin conjugated cocktail of antibodies to CD43, CD4, CD8, CD11b, CD11c, Gr-1, CD138, CD38 and IgD; followed by magnetic bead-depletion of labeled cells, as previously described.^12^^,^^17^^,^^18^ This resulted in GC B cell purity ≥ 95%.

Purified GC B cells were warmed to 37 °C with 5% CO_2_ in B cell medium (RPMI-1640 medium supplemented with 10% FBS, penicillin/streptomycin, glutamine and 50 μM β-mercaptoethanol) for 30 min and stimulated with 200 ng/ml NP-Ficoll (LGC Biosearch Technologies) and 5 μg/ml anti-CD40 antibody tetramer for 3.5 hours. The stimulation is to progress GC B cells into cell cycle for efficient retroviral transduction. Anti-CD40 antibody tetramers were made by incubating biotinylated anti-CD40 antibody (FGK45, Bio X Cell or prepared in our lab) with streptavidin at 5:1 molecular ratio.

*Pten* shRNA (shPTEN.1522^19^) and shCD8 were cloned into the MSCV-based retroviral vector pLMPd (gift of Shane Crotty^20^). Retroviruses were produced using the Plat-E cell line. Stimulated GC B cells were transduced with the retroviral vectors by centrifugation at 800× *g* for 2 h at 32 °C using virus-containing supernatant. For GC B cell transfer experiments, cells were washed immediately after transduction and transferred into day 5-immunized B1-8i mice via i.v. injection. For in vitro studies, the transduced cells were incubated at 37 °C with anti-CD40 tetramer. After 24 h, when shRNA expression was established, 10 ng/mL IL-21 (PeproTech or BioLegend) was added to the culture. Cells were analyzed by flow cytometry 1–2 d later.

### Measurement of PIP3 abundance following CD40 stimulation

Isolated B1-8i naïve B cells and GC B cells were stimulated with anti-CD40 tetramer, and 10^6^ cells were collected per time point. Samples were processed per the manufacturers’ instructions included in the Echelon Biosciences Mass ELISA kits for PIP3 (K-2500s). The ELISA assays were measured at 450 nM on a Molecular Devices SpectraMax i3 plate reader. The standard curve was fit assuming a sigmoidal dose-response with variable slope and the amount of phosphatidylinositol in each sample was interpolated using the GraphPad Prism software package.

### Flow cytometry staining, antibodies, and analysis

The dLNs (both inguinal lymph nodes) or Peyer’s patches were taken and processed into single cells with frosted glass slides, then filtered using 70 µm strainers (Corning). Cells were stained with Ghost Dye Violet 510 (Cytek Cat: SKU 13-0870-T100). The cells were then washed and blocked with anti-CD16/32 antibody (S17011E, BioLegend Cat.no. 156604, clone: S17011E) prior to staining with fluorochrome-conjugated antibodies to: B220 (PerCP-Cy5.5, BioLegend Cat: 103236, clone: RA3-6B2), CD95 (PE-Cy7, BD Cat: 557653, clone: Jo2), CD38 (BV711, BD Cat: 740697, clone: 90), CD38 (A647, BD Cat: 562769, clone: 90), CD138 (BV605, BioLegend Cat: 142531, clone: 281-2), CD138 (BV650, BioLegend Cat: 142518, clone: 281-2), CD44 (APC-Cy7, BioLegend Cat: 103028, clone: IM7), CXCR4 (BV421, BioLegend Cat: 146511, clone: L276F12), CD86 (BV785, BioLegend Cat: 105043, clone: GL1), CD86 (BUV395, BD Cat: 564199, clone: GL1), CCR6 (BV605, BioLegend Cat: 129819, clone: 29-2L17), IgG1 (APC/Fire750, BioLegend Cat: 406624, clone: RMG1-1), CD45.1(PE, Tonbo Cat: 50-0453-U100, clone: A20) CD45.2(A488, made in lab, clone: 104), IgM (biotin, SouthernBiotech #1021-08, polyclonal), IgA (PE, SouthernBiotech Cat: 1040-09, polyclonal), Streptavidin (BV421, BD Cat. no. 563259). Surface staining was incubated for 20 min on ice followed by fixation, as described below.

For intracellular staining: after surface staining, cells were fixed with 2% PFA for 20 min at room temperature. The cells were washed and permeabilized in Triton-permeabilization buffer (PBS with 2% FCS, 0.02% Azide, 2 mM EDTA and 0.1% Triton X-100) for 20 min at room temperature. The cells were then stained with antibodies to: IRF4 (A488, BioLegend Cat.no. 646406, clone: IRF4.3E4), IRF4 (PE, BioLegend Cat: 646404, clone: IRF4.3E4), BLIMP1 (PE-CF594, BD Cat: 564269, clone 5E7), BLIMP1 (Al647, BioLegend Cat. no. 150004, clone 5E7), PTEN (Cell Signaling Technology, Cat: 9188, clone: D4.3), anti-Rabbit 2nd antibody (Cy3 conjugated, BioLegend Cat: 406402).

For experiments using inhibitors, cells were treated with 10 μM PTEN inhibitor (SF1670; Calbiochem) dissolved in DMSO or the same amount of DMSO for vehicle control, as described in the figure legends, before CD40 stimulation. Cells were then fixed and permeabilized in saponin-based Perm/Wash buffer (Cat. no. 554723, BD Biosciences) supplemented with 1.5% paraformaldehyde (PFA) and blocked with Fc receptor antibody (clone: 2.4G2, prepared in the lab) prior to staining with fluorochrome conjugated antibodies: Phospho-S6 (Ser235/236; clone: D57.2.2E, Cell Signaling Technology), B220 (clone: RA3-6B2, BD Bioscience), PNA (Vector Lab), CD38 (clone: 90, prepared in our lab), CD95 (clone: Jo2, BD Biosciences).

To stain for p-STAT3 (Y705), after fixation in PFA, cells were permeabilized with ice cold methanol added dropwise to the cell pellet while vortexing gently, then cells were left in methanol for 30 min on ice. Cells were then washed with staining buffer and blocked with Fc receptor antibody, followed by staining for 1 h at 4C with the following antibodies and reagents: p-Stat3 (A647, pY705; BD Cat: 562071, clone: 4/P-STAT3), PNA (A488, Invitrogen Cat: L21409), B220 (PerCP-Cy5.5, BioLegend Cat: 103236, clone: RA3-6B2), CD95 (PE-Cy7, BD Cat: 557653, clone: Jo2), CD138 (BV650, BioLegend Cat: 142518, clone: 281-2), IgG1 (APC/Fire750, BioLegend Cat: 406624, clone: RMG1-1).

Stained cells were analyzed on an LSRII or Fortessa flow cytometer. Data were analyzed with FlowJo 10 software. GC B cells were gated as B220^+^ CD95^+^ CD38^-^ (or PNA^+^) cells, and non-GC B cells were gated as B220^+^ CD38^+^ CD95^-^ cells.

### Statistical analysis

Statistical analyses were conducted using Prism 10 software (GraphPad Software). For comparing 2 groups, *P*-values were determined using Student *t*-tests or paired Student *t*-tests (2-tailed) as defined in the figure legends; for comparing more than 2 groups, 1-way ANOVA followed by Tukey’s test was applied. Differences between groups were considered significant for *P* values < 0.05 (*P < 0.05; **P < 0.01; ***P < 0.001; ****P < 0.0001).

## Results

### Deleting *Pten* in established GC B cells using the hCD20^TamCre^ allele

**Figure 1.**
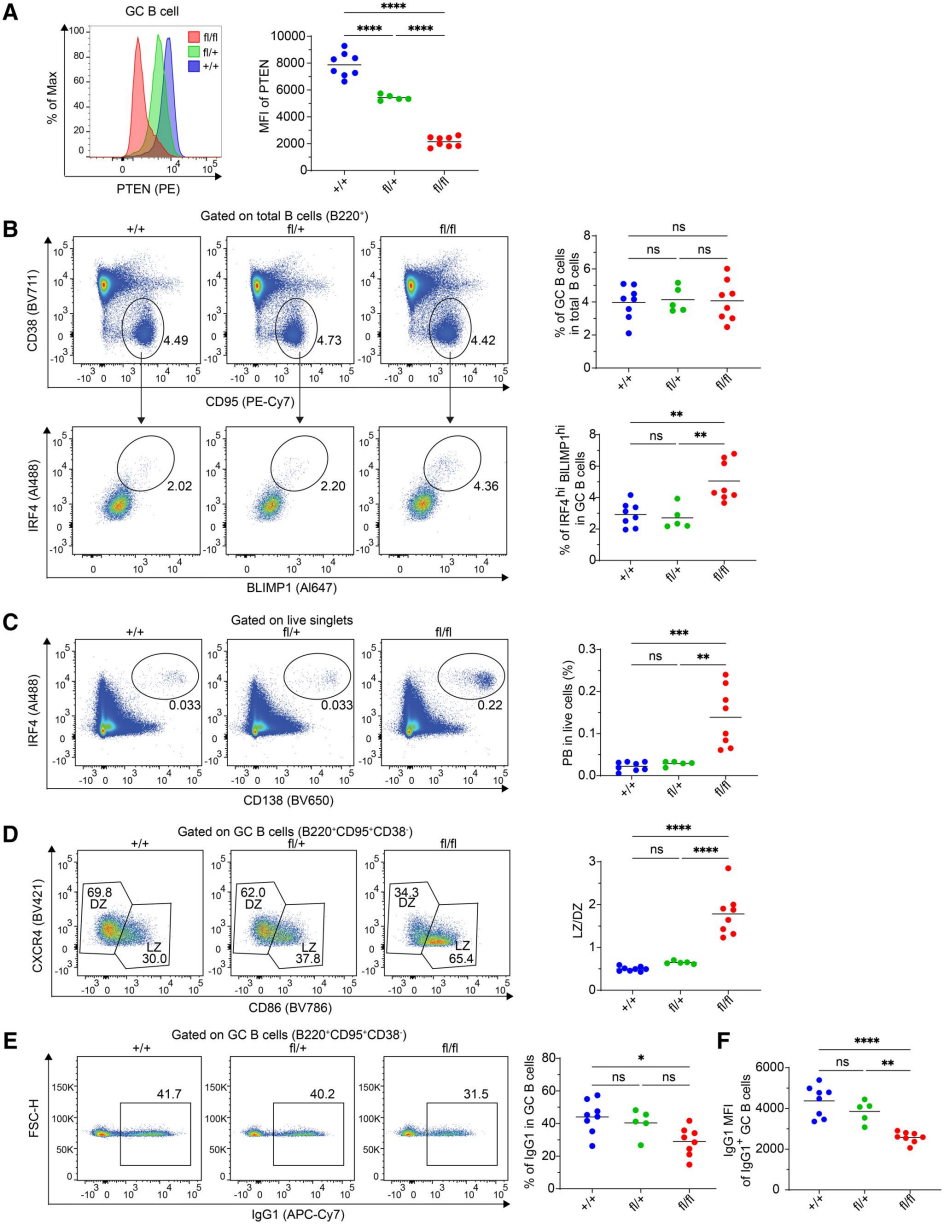
*Pten* deletion in established GC B cells promotes PC effector differentiation. *Pten*^fl/fl^ hCD20^TamCre^ (“fl/fl”), *Pten*^fl/+^ hCD20^TamCre^ (“fl/+”), *Pten*^+/+^ hCD20^TamCre^ (“+/+”) mice were s.c. immunized with NP-OVA adjuvanted with AddaVax on day 0. On day 7, a single dose of tamoxifen was administered, and dLNs were analyzed by FACS on day 14. (A) Representative histogram of intracellular staining of PTEN and statistical analysis of PTEN MFI (median fluorescence intensity). (B) Representative flow plots and statistical analysis of GC B cells and PC precursors. (C) Representative flow plots and statistical analysis of PCs. (D) Representative flow plots and statistical analysis of the LZ/DZ frequency ratio of GC B cells. (E) Representative flow plots and statistical analysis of IgG1^+^ GC B cells. Date are combined from two independent experiments with 5 to 8 mice in each group. Each dot represents a single mouse. Statistical significance was determined by one-way ANOVA followed by Tukey’s multiple comparisons test (*P ≤ 0.05; **P ≤ 0.01; ***P ≤ 0.001; ****P ≤ 0.0001). ns, not significant (*P* > 0.05).

The GC phenotypes elicited in previous studies utilizing CD19- or Cγ1-Cre mediated PTEN deletion could have been due to the impact of *Pten* in B cells before GC formation.^6–8^ To investigate the function of PTEN in ongoing GC B cells, we crossed the *Pten*^fl/fl^ mice with the hCD20^TamCre^ mice, generating *Pten*^fl/fl^ hCD20^TamCre^ mice. This approach allows for the inducible deletion of *Pten* in B cells through tamoxifen treatment after GC formation. To confirm the efficiency of *Pten* deletion and its effects on PTEN expression, we subcutaneously (s.c.) immunized *Pten*^fl/fl^ hCD20^TamCre^, *Pten*^fl/+^ hCD20^TamCre^ and control (*Pten^+/+^* hCD20^TamCre^) mice with NP-OVA. On day 7 post-immunization, when the GC reaction was fully established, a single dose of tamoxifen was administered to induce *Pten* deletion. Since PTEN has a relatively long half-life,^21^ we began our analysis of mice 7 d after tamoxifen administration to allow sufficient time for PTEN decay and the manifestation of its effects. First, we performed intracellular flow cytometry on the draining lymph nodes (dLNs) to assess PTEN expression levels. Our data confirmed that PTEN is highly expressed in GC B cells compared to non-GC B cells (mostly naïve B cells) gated in the same flow staining reaction, and further demonstrated that a single dose of tamoxifen can efficiently reduce PTEN protein levels in GC B cells 7 d after tamoxifen administration in the conditional knockout (cKO) mouse lines (Fig. 1A). This reduction was more pronounced in GC B cells than in non-GC B cells (Fig. 1A and Fig. S1A), likely due to the relatively long half-life of PTEN protein;^21^ the rapid division of GC B cells presumably would accelerate protein depletion following gene deletion.

### *Pten* deletion in established GC B cells promotes effector differentiation to PC

To examine whether the deletion of *Pten* after the formation of GCs impacts GC B cell functionality, we immunized mice s.c. with NP-OVA adjuvanted with AddaVax and administered tamoxifen 7 d later. Analyses were performed 7 d post-tamoxifen treatment. Acute PTEN ablation after GC formation did not affect the maintenance of GC B cells (Fig. 1B). This finding contrasts with prior studies that used the CD19- or Cγ1-Cre systems,^6^^,^^8^ which found alterations in GCs. Since we engineered complete deletion of PTEN protein in established GC B cells (Fig. 1A), these differences between studies likely reflect PTEN functions at earlier time points to affect GC entry, rather than to affect cells that have already differentiated into GC. We then explored whether PTEN plays a role in regulating the effector differentiation of established GC B cells, a question not addressed in earlier studies. Acute *Pten* deletion significantly increased the proportion of GC B cells with a PC precursor phenotype (IRF4^hi^BLIMP1^hi^, Fig. 1B).^18^^,^^22–24^ In contrast, *Pten* deletion had no effect on the proportion of MBC precursors (CCR6^hi^ IRF4^lo^) among GC B cells (Fig. S1B). Consistent with effects on GC positive PC precursors, we observed a significant increase in CD138-expressing PCs in *Pten*^fl/fl^ hCD20^TamCre^ mice compared to controls (Fig. 1C). These findings thus reveal a previously unrecognized role of PTEN in negatively regulating the effector differentiation of GC B cells toward PCs.

Induced PTEN deletion after GC establishment also markedly altered the zonal distribution of GC B cells, leading to an increased light zone (LZ) to dark zone (DZ) ratio (Fig. 1D). This is likely due, in the absence of PTEN, to enhanced signaling down the PI3K-AKT-FOXO1 pathway. Enhanced signaling would reduce nuclear FOXO1, which is in turn required for DZ formation and is thought to be coupled with isotype switching in the GC.^25^^,^^26^ Surprisingly, despite significant a reduction of the DZ size, PTEN deletion only modestly reduced the proportion of IgG1-expressing GC B cells (Fig. 1E). This phenotype also contrasts with previous studies in which PTEN deletion at earlier stages of B cell development nearly ablated IgG1 class-switching.^6–8^ Notably, when analyzing IgG1^+^ GC B cells, PTEN deficiency resulted in a significant reduction in surface immunoglobulin (Ig) expression (Fig. 1F), suggesting that PTEN activity may support BCR levels in GC B cells, likely by modulating signaling^12^ that would otherwise result in receptor internalization and downregulation.^27^

### Tracking PTEN-deficient GC B cells in vivo using a transfer model

**Figure 2.**
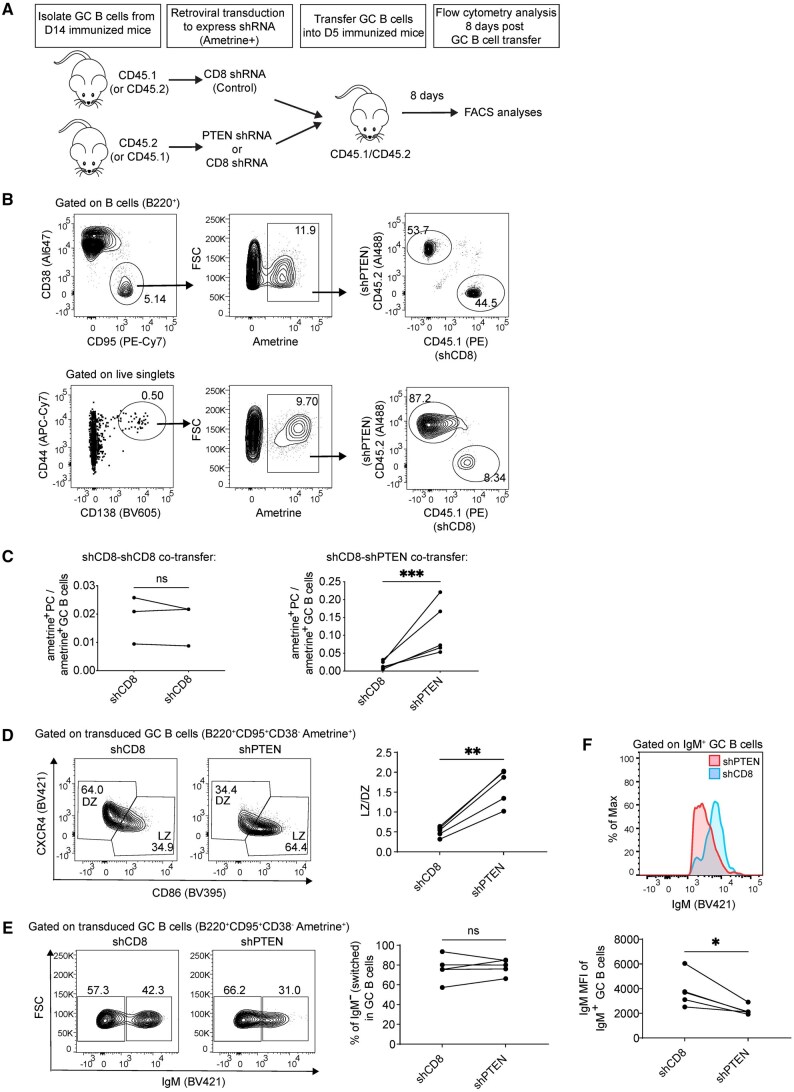
Tracking PTEN-deficient GC B cells in vivo using a transfer model. (A) Schematic representation of the procedure for the GC B cell transduction-transfer model. (B) Following the scheme shown in (A), spleens were analyzed by flow cytometry 8 d after GC B cell transfer. Plots depict the relative abundance of shCD8- and shPTEN-transduced GC B cells, as well as GC B cell-derived PCs in the same mice. (C) Statistical comparisons were performed to analyze the ratio of PCs to GC B cells within shRNA viral vector-transduced populations for both the shCD8-shCD8 co-transfer and shCD8-shPTEN co-transfer models. (D) Analysis of the light zone (LZ) and dark zone (DZ) distribution in the shCD8-shPTEN co-transfer models. (E) Analysis of the proportion of switched versus unswitched GC B cells in the shCD8-shPTEN co-transfer models. (F) Comparison of IgM MFI of IgM^+^ GC B cells as gated in (E). Data are from two independent experiments, with 3 or 5 recipient mice as shown. Statistical significance was determined by two-tailed unpaired *t*-test (*P ≤ 0.05; **P ≤ 0.01; ***P ≤ 0.001). ns, not significant (*P* > 0.05).

While timed administration of tamoxifen deletes *Pten* in established GC B cells, some B cell precursors could also be affected and could have partially contributed to the phenotypes observed, including PCs generated, which could have emanated from GC or non-GC precursors. Given the contrast between our results and prior studies using constitutive Cre’s, this seems unlikely to explain our findings. Nonetheless, to overcome this limitation, and to validate the GC B cell intrinsic role of PTEN in regulating PC development, we developed a GC B cell co-transfer system. This allowed us to modify gene expression directly in established GC B cells, then to track the fate of PTEN-deficient and control GC B cells and their progeny in the same mice, using CD45 congenic markers. We purified day 14 GC B cells from CD45.1 and CD45.2 mice and transduced them with retroviral vectors expressing either CD8 shRNA as control (“shCD8”) or PTEN shRNA (“shPTEN”, Fig. 2A**)**, along with ametrine to report on transduction. shPTEN transduction effectively knocked down PTEN expression in B cells (Fig. S2A). Immediately after transduction, we mixed equal numbers of CD45.1 and CD45.2 donor GC B cells and transferred them into day 5-immunized CD45.1/CD45.2 recipient mice. Eight days later, we analyzed transferred GC B cells and the PCs derived from them by flow cytometry.

PTEN knockdown had a minimal impact on the frequency of GC B cells. However, PCs derived from transduced GC B cells were predominantly shPTEN (Fig. 2B), likely due to enhanced differentiation of PC precursors within GC B cells following PTEN knockdown (Fig. S2B). To assess the efficiency of GC B cell-to-PC differentiation, we calculated the ratios of PC to GC B cell numbers within transduced cells expressing ametrine. In the control shCD8-shCD8 co-transfer, the PC/GC B cell ratios among ametrine^+^ cells were similar. However, in shCD8-shPTEN co-transferred mice, shPTEN transduction significantly increased the PC/GC B cell ratios compared to shCD8 transduction (Fig. 2C). These data confirm in an independent system our results using the *Pten*^fl/fl^ hCD20^TamCre^ approach, indicating that PTEN deficiency indeed can promote PC development from established GC B cells in a cell-intrinsic manner. PTEN knockdown in the GC B cell co-transfer model also elevated the LZ to DZ ratio (Fig. 2D). However, PTEN knockdown did not affect the class-switched/IgM ratio in GC B cells (Fig. 2E), indicating that PTEN knockdown in established GC B cells reduces the DZ without acutely impacting class switching. Similar to the case with IgG1 in the Cre-mediated deletion system (Fig. 1F), when analyzing IgM^+^ GC B cells, PTEN knockdown also resulted in a significant reduction in surface Ig expression (Fig. 2F).

### PTEN regulates Peyer’s patch GC B cell homeostasis and differentiation

**Figure 3.**
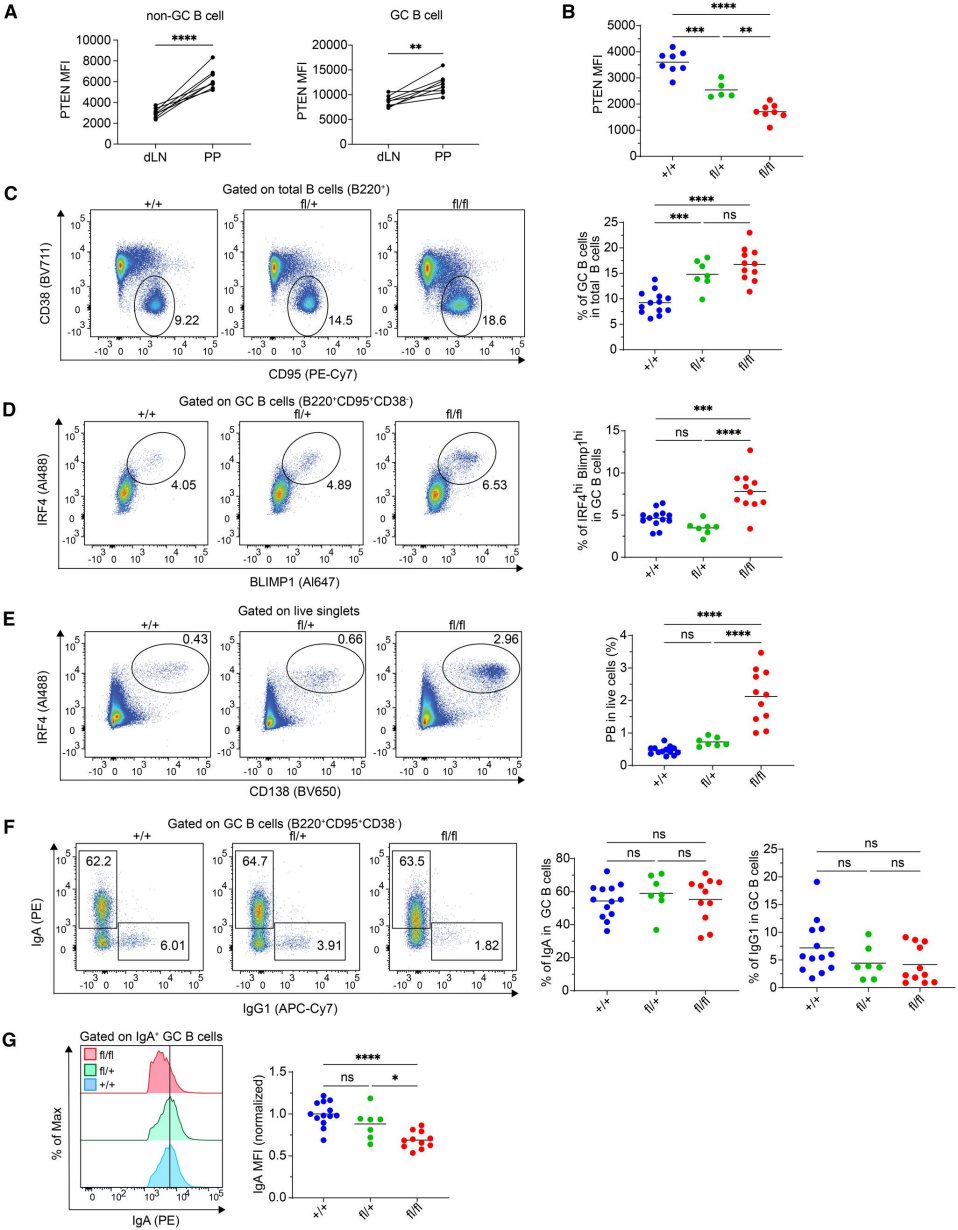
*Pten* deletion expends Peyer’s patch GC B cells and promotes their differentiation to PCs. (A) Mice were s.c. immunized with NP-OVA adjuvanted by AddaVax. Day 7 post immunization, FACS analyses were performed to compare PTEN expression levels between dLN GC B cells induced by immunization and chronic GC B cells from Peyer’s patches of the same mice. (B) Validation of *Pten* deletion efficiency in *Pten*^fl/fl^ hCD20^TamCre^ (“fl/fl”) mice 7 d post a single dose of tamoxifen treatment. Data shown are PTEN MFI examined by FACS. (C) Representative flow plots and statistical analysis of GC B cells. (D) Representative flow plots and statistical analysis of PC precursors within GC B cells. (E) Representative flow plots and statistical analysis of PCs. (F) Representative flow plots and statistical analysis of IgA and IgG1^+^ isotype expression among GC B cells. (G) Representative histogram and statistical analysis of IgA MFI among IgA^+^ GC B cells. Data are combined from three independent experiments with 7 to 13 mice in each group. Each dot is a single mouse. Statistical significance was determined by one-way ANOVA followed by Tukey’s multiple comparisons test (*P ≤ 0.05; **P ≤ 0.01; ***P ≤ 0.001; ****P ≤ 0.0001). ns, not significant (*P* > 0.05).

In contrast to the newly formed GCs induced by immunization in dLNs, gut-associated lymphoid tissues (GALT), such as Peyer’s patches (PPs), typically maintain a persistent GC response with a high proportion of IgA GC B cells. These IgA GC B cells display unique signaling characteristics compared to other isotypes; for instance, IgA BCR transmits stronger signals, whereas BCR signaling in other GC B cell isotypes tends to be more attenuated.^1^^,^^28^ Interestingly, we observed that both GC B cells and non-GC B cells (mainly naïve B cells) from PPs exhibited higher PTEN expression levels than their counterparts elicited after immunization in dLNs (Fig. 3A).

Building on this finding, we questioned whether PTEN might play different roles in regulating PP GC B cells. To explore this, we used the same *Pten* conditional deletion model as in Fig. 1 and analyzed PP GC B cells 7 d after a single dose of tamoxifen. We first verified that tamoxifen treatment reduced PTEN protein levels in PP GC B cells in *Pten*^fl/+^ hCD20^TamCre^ and *Pten*^fl/fl^ hCD20^TamCre^ mice (Fig. 3B). Interestingly, unlike in dLN GCs, induced PTEN deficiency in spontaneous PP GC B cells led to an expansion of GC B cells that correlated with the extent of reduction of PTEN protein levels (Fig. 3C). These results indicate that PTEN plays a critical role in negatively regulating PP GC B cell expansion and homeostasis in a dose dependent manner, reminiscent of the role of PTEN in negatively regulating some GC-type diffuse large B cell lymphomas.^13^ Consistent with dLN GCs, PTEN deletion in PP GC B cells also enhanced their differentiation into PCs, as evidenced by an increase in PC precursors among GC B cells and an overall increase in PCs (Fig. 3D, E). PTEN deficiency did not affect the percentage of IgA^+^ cells within PP GC B cells (Fig. 3F). While a trend toward reduced IgG1 was observed after PTEN deletion, this change was not statistically significant (Fig. 3F). Finally, we noted a significant reduction in surface IgA levels in PTEN-deficient PP GC B cells (Fig. 3G), similar to the effect on IgG1 and IgM expressions seen in dLN (Figs 1F and 2F). These findings indicate that PTEN regulates ongoing GC B cell reactions within GALT in a manner distinct from the newly formed GC B cells in the dLNs.

### PTEN activity reprograms CD40 signaling in GC B cells

**Figure 4.**
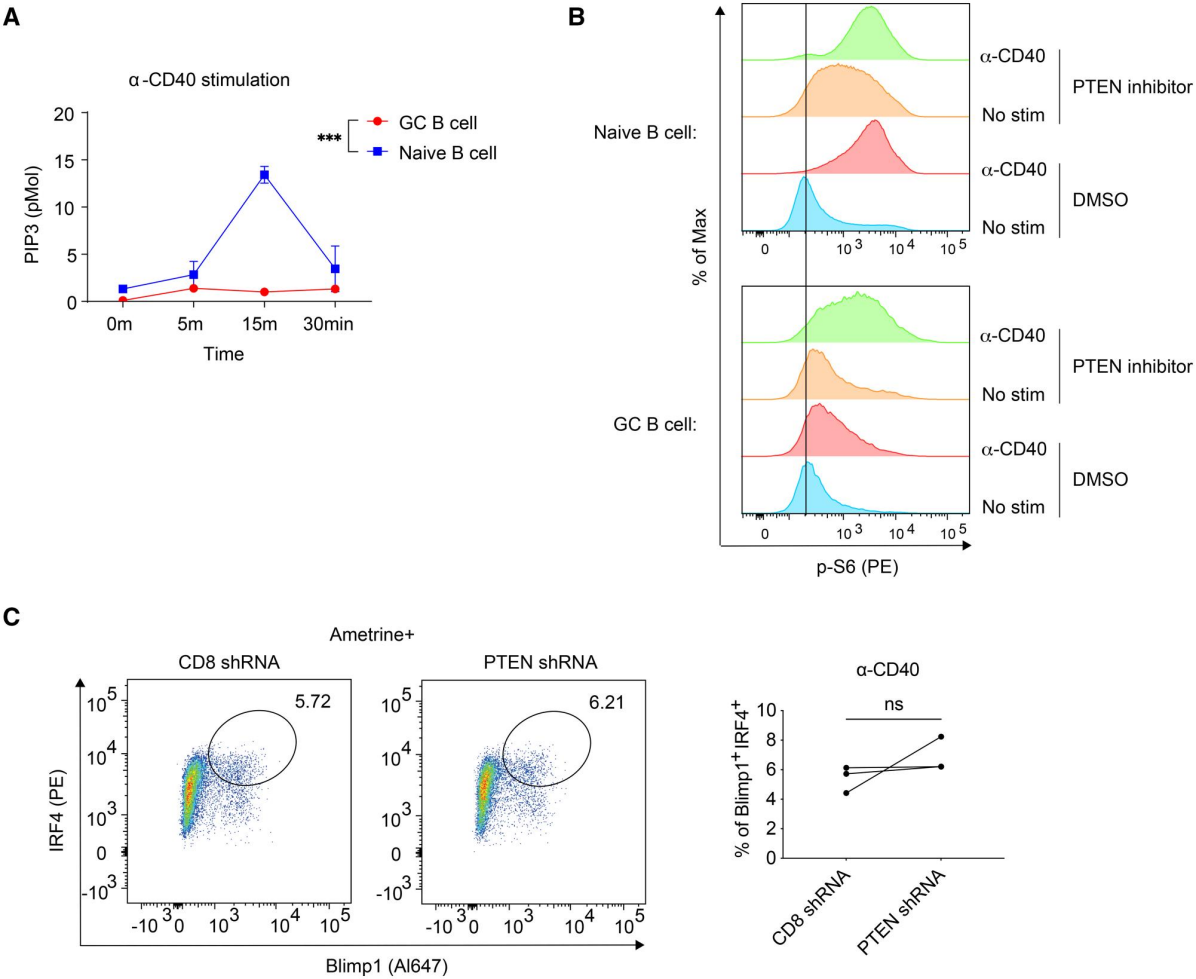
PTEN activity mediates CD40 signaling reprogramming in GC B cells. (A) Purified B1-8i naïve and GC B cells were stimulated with anti-CD40 tetramer. Inositol lipid PIP3 was measured by ELISA of cell lysates. Data represent two experiments with cells pooled from 3 to 5 mice in each experiment. (B) Splenocytes from NP-CGG immunized B1-8i mice were treated with DMSO or PTEN inhibitor (SF1670) for 30 min before CD40 stimulation with anti-CD40 tetramer. 30 minutes after stimulation, cells were then fixed and analyzed by flow cytometry for p-S6. (C) Purified B1-8i GC B cells were transduced with either the shCD8 or shPTEN viral vector and then returned to culture under anti-CD40 stimulation for 2–3 d. Cells were then analyzed for Blimp1 and IRF4 expression by FACS for PC differentiation. Data represent three samples for each group from two independent experiments. Statistical significance was determined by 2-way ANOVA (A) and 2-tailed paired *t*-test (C). ***P ≤ 0.001; ns, not significant (*P* > 0.05).

In both dLN and PP GC B cells, PTEN functions to restrict their differentiation into PCs. To understand the underlying mechanisms, we investigated how PTEN regulates CD40 and IL-21R signaling in GC B cells, as these signals cooperate to drive GC B cell differentiation into PCs.^18^^,^^29^ We previously demonstrated that CD40 signaling in GC B cells is reprogrammed to selectively activate the NF-κB pathway but not the AKT-mTORC1 pathway; whereas in naïve B cells, CD40 stimulation can activate both pathways.^17^ To assess whether PTEN phosphatase activity plays a role in this reprogramming, we first measured PtdIns(3,4,5)P3 (PIP3) levels, the direct substrate of PTEN, following CD40 stimulation in both GC and naïve B cells. In naïve B cells, CD40 stimulation led to an upregulation of PIP3 levels, which peaked at 15 min. In contrast, PIP3 levels barely changed in GC B cells after CD40 stimulation, likely due to the high levels of PTEN in these cells (Fig. 4A). To further test whether PTEN was responsible for blunted PIP3 generation following CD40 ligation in GC B cells, we pretreated the cells with the PTEN phosphatase inhibitor SF1670 for 30 min, followed by CD40 stimulation, and then examined S6 phosphorylation that is in turn dependent on the AKT-mTORC1 pathway activated by PIP3.^12^^,^^30^ Indeed, commensurate with the lack of PIP3 generation and as we previously reported, GC B cells generate little p-S6 compared to naïve B cells after CD40 ligation. Critically, PTEN inhibition rescued S6 phosphorylation in GC B cells following CD40 signaling, indicating that PTEN activity is essential for reprogramming CD40 signaling in GC B cells (Fig. 4B). Next, we asked whether PTEN inhibition alone is sufficient to drive GC B cell differentiation toward PCs following CD40 signaling. Due to cell toxicity of the PTEN inhibitor in long-term cultures, we used PTEN shRNA to knock down PTEN in GC B cells (as in Fig. 2) and cultured the cells for an additional 2 d with CD40 antibodies. However, we observed that CD40 signaling alone was insufficient to strongly induce PC differentiation from GC B cells, regardless of PTEN levels (Fig. 4C).

### PTEN deficiency prolongs IL-21R signaling in GC B cells, enhancing PC differentiation upon IL-21R and CD40 co-stimulation

**Figure 5.**
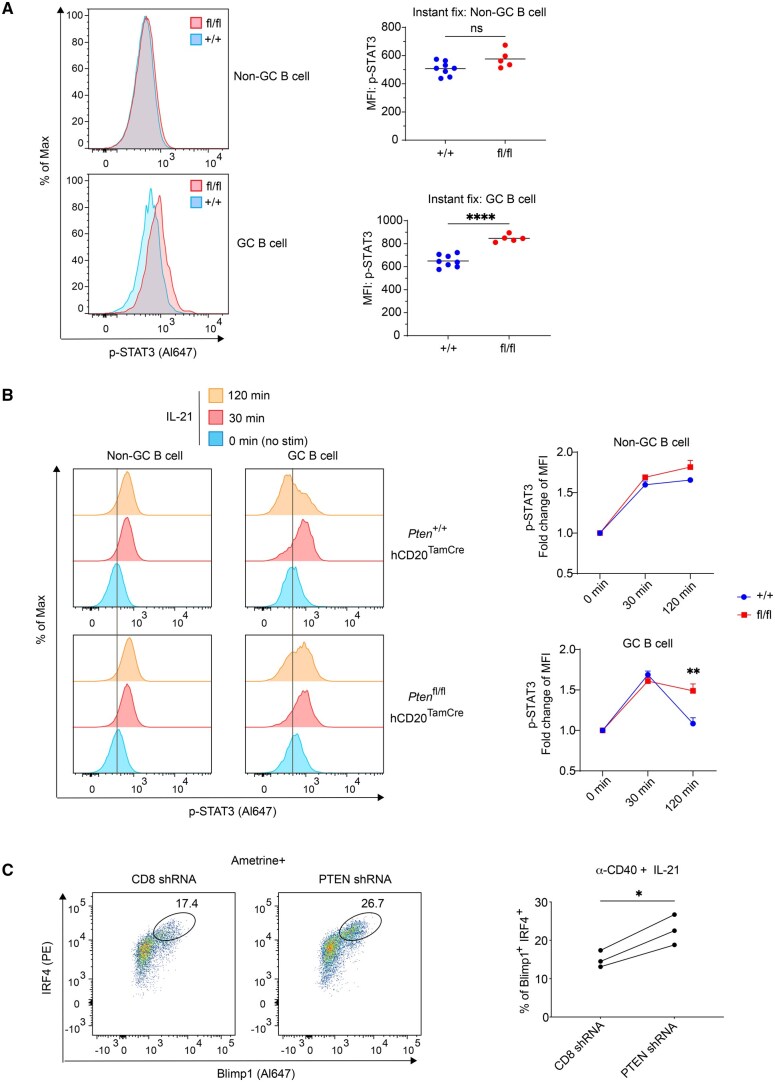
PTEN restrains IL-21R signaling in GC B cells and limits their differentiation to PCs. *Pten*^fl/fl^ hCD20^TamCre^ (“fl/fl”) and *Pten*^+/+^ hCD20^TamCre^ (“+/+”) mice were s.c. immunized with NP-OVA adjuvanted with AddaVax on day 0. On day 7, a single dose of tamoxifen was administered, and dLNs were analyzed by FACS 4 d post tamoxifen treatment. (A) Cells from the dLNs were instantly fixed and analyzed by FACS to compare basal level of p-STAT3. +/+, n = 8; fl/fl n = 5 from three experiments. (B) Cells from the dLNs were rested in a 37 °C incubator for 30 minutes and then stimulated with 20 ng/ml IL-21 for 0, 30, and 120 min. Cells were analyzed by FACS for p-STAT3 after stimulation. +/+, n = 4; fl/fl n = 7 from three experiments. (C) Purified B1-8i GC B cells were transduced with either the shCD8 or shPTEN viral vector and then returned to culture under IL-21 and anti-CD40 stimulation for 2–3 d. Cells were then analyzed for Blimp1 and IRF4 expression by FACS for PC differentiation. Data represent 3 samples for each group from 2 independent experiments. Statistical significance was determined by 2-tailed unpaired *t*-test (A), 2-way ANOVA followed by Tukey’s multiple comparisons test (B), and 2-tailed paired *t*-test (C). (*P ≤ 0.05; **P ≤ 0.01; ****P ≤ 0.0001). ns, not significant (*P* > 0.05).

We previously found that CD40 signals cooperate with IL-21R signals to promote GC B cell differentiation into PCs.^18^ This work also revealed that IL-21R signaling is reprogrammed in GC B cells compared to naïve B cells: IL-21R stimulation led to sustained phosphorylation of STAT3 (Y705) in naïve B cells but not in GC B cells, despite strong STAT3 phosphorylation at early time points in both cell types.^18^ We hypothesized that PTEN in GC B cells may influence PC differentiation by modulating both CD40 and IL-21R signaling. To investigate this, we used the *Pten*^fl/fl^ hCD20^TamCre^ mice to delete PTEN in established GC B cells. Interestingly, acute PTEN deletion (4 d post-tamoxifen treatment) led to an increase in basal p-STAT3 (Y705) levels in GC B cells (Fig. 5A). To specifically examine IL-21R signaling, we rested dLN cells from immunized control and *Pten*-deleted mice, and then stimulated them with IL-21 for 0, 30, and 120 min, followed by FACS analyses of p-STAT3 (Y705). Consistent with previous findings,^18^ IL-21 stimulation resulted in more persistent p-STAT3 (Y705) in naïve B cells from 30 to 120 min, whereas this phosphorylation was transient in GC B cells from the control mice (Fig. 5B). Strikingly, PTEN deficiency substantially prolonged the duration of IL-21-induced p-STAT3 (Y705) in GC B cells, leading to significantly higher p-STAT3 levels 2 h after IL-21R stimulation (Fig. 5B). These findings highlight an important role of PTEN in reprogramming IL-21R signaling in GC B cells by limiting the duration of downstream STAT3 phosphorylation.

To test whether PTEN regulation of IL-21R signaling is connected to enhanced PC differentiation from GC B cells when PTEN itself is reduced, we isolated primary GC B cells and transduced them with either shPTEN or shCD8 (control) retroviruses. We then cultured them under CD40 and IL-21R stimulation conditions for 2 d, followed by FACS analysis. Indeed, when both CD40 and IL-21R were stimulated, PTEN knockdown significantly enhanced GC B cell differentiation into PC (Fig. 5C). Notably, CD40 stimulation alone (Fig. 4C) did not promote such differentiation, and we previously showed that IL-21 signals alone have no potential to enhance PC differentiation in unmanipulated GC B cells.^18^ Hence, these results, obtained under defined stimulation conditions with PTEN knockdown, highlight the important role of PTEN in regulating GC B cell differentiation into PCs through its modulation of the combination of IL-21R and CD40 signaling.

## Discussion

Our previous work revealed that GC B cells express significantly higher levels of PTEN protein compared to naïve B cells.^12^ In this study, we addressed a critical knowledge gap regarding the role of PTEN in regulating established GC B cells. We discovered that PTEN reprograms signaling through both the CD40 and IL-21 receptors in GC B cells. While the PI3K-AKT pathway downstream of CD40 signaling is a well-established target of PTEN regulation, our findings that PTEN specifically controls the duration of IL-21R signaling induced p-STAT3 in GC B cells are unexpected. The canonical activation of the PI3K-AKT pathway, which is negatively regulated by PTEN, is not typically linked to the phosphorylation of STAT3. However, recent studies suggest that PI3K may enhance STAT3 phosphorylation through TEC kinase in certain cancer cells.^31^ PTEN is known to have dual lipid and protein phosphatase activities,^32^ and it is also possible that its protein phosphatase function contributes directly or indirectly to the shortened duration of STAT3 activation following IL-21R stimulation. Although the precise mechanisms connecting PTEN and the IL-21R-STAT3 pathway remain unclear and need to be further investigated, our findings show that high PTEN expression in GC B cells acts as a brake on their differentiation into PCs. This regulatory mechanism may facilitate affinity-based selection and safeguard against autoimmunity by preventing premature or autoimmune PC development from GC. Similar mechanisms may also operate in regulating transformed GC B cells during lymphomagenesis.

mTORC1 activation in GC B cells is initiated by T cell-derived signals in the LZ, promoting their subsequent proliferation in the DZ.^33^ In PTEN-deficient GC B cells, CD40 signaling induces stronger mTORC1 activation; however, this is paradoxically accompanied by reduced DZ-to-LZ ratios. This likely results from enhanced inactivation of FOXO1—another downstream target of the PTEN-AKT pathway—which counteracts the DZ proliferation typically supported by mTORC1. This aligns with the essential role of FOXO1 in directing the DZ program in GC B cells.^25^^,^^26^ Following positive selection in the LZ, GC B cells receiving CD40 and IL-21R signals can initiate PC differentiation. mTORC1 is known to facilitate this process.^34^^,^^35^ Although CD40 signaling robustly activates mTORC1 in PTEN-deficient GC B cells, IL-21R signaling remains necessary for full effector differentiation into PCs. This reflects the critical role of IL-21R for optimal IRF4 expression, as demonstrated in our previous study.^18^ Together, these findings indicate that while PTEN modulates both CD40 and IL-21R signaling, the availability of T cell-derived IL-21 may still restrict PC differentiation in vivo, even in the absence of PTEN. Thus, the LZ/DZ phenotype in PTEN deficiency integrates both input signals (such as T cell help) and output fate decisions (such as proliferation or differentiation).

Previous studies suggest that IgA GC B cells in PPs exhibit stronger intracellular signaling and greater resistance to cell death, which may contribute to the IgA response against the microbiome.^28^ Intriguingly, we observed that the spontaneously elicited GC B cells from PPs express higher levels of PTEN compared to dLN GC B cells induced by immunization. This raises the possibility of potential interactions between BCR signaling and PTEN expression. For instance, enhanced IgA BCR signaling could potentially drive cumulative PTEN expression; reciprocally, elevated PTEN may serve to better control signaling from IgA BCR. Indeed, acute PTEN reduction resulted in expansion of PP, but not dLN, GC B cells. Further research is needed to understand if this PTEN-mediated regulatory pathway is essential for maintaining immune tolerance to the microbiome, food or self-antigen during chronic GC responses in mucosa-associated lymphoid tissue.

## Supplementary Material

vkaf160_Supplementary_Data

## Data Availability

The data underlying this article are available in the article and in its online supplementary material.
