# Supplementary material for: PTEN restricts IL-21R signaling in GC B cells and suppresses their differentiation to plasma cells
Source: J Immunol. 2025 Aug 7;214(11):2936–46. doi: 10.1093/jimmun/vkaf160 (PMC12646060; doi:10.1093/jimmun/vkaf160)
Supplement: vkaf160_Supplementary_Data [file vkaf160_supplementary_data.pdf]

Fig. S1

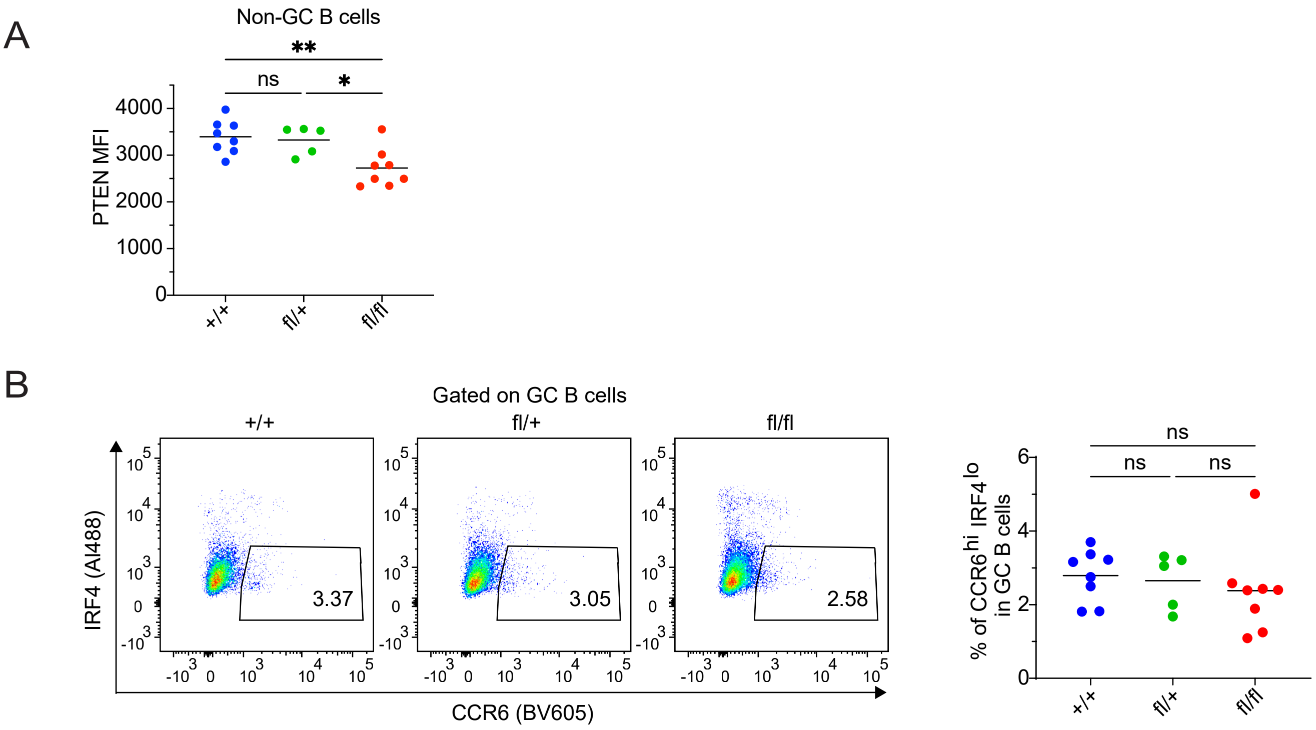

Fig. S1 | PTEN expression levels in non-GC B cells and the impact of *Pten d*eletion on GC B cell differentiation to memory B cell (MBC) precursors. *Pten*$^{fl/fl}$ hCD20^{TamCre} ("fl/fl"), *Pten*$^{fl/+}$ hCD20^{TamCre} ("fl/+"), *Pten*$^{+/+}$ hCD20^{TamCre} ("+/+") mice were immunized with NP-OVA adjuvanted with AddaVax on day 0. On day 7, a single dose of tamoxifen was administered, and dLNs were analyzed by FACS on day 14. (**A**) PTEN MFI of non-GC B cells. (**B**) Representative flow plots and statistical analysis of MBC precursors within GC B cells. All data represent two independent experiments with 5 to 8 mice in each group. Statistical significance was determined by one-way ANOVA followed by Tukey's multiple comparisons test ($*P \leq 0.05$; $**P \leq 0.01$). ns, not significant ($P > 0.05$).

Fig. S2

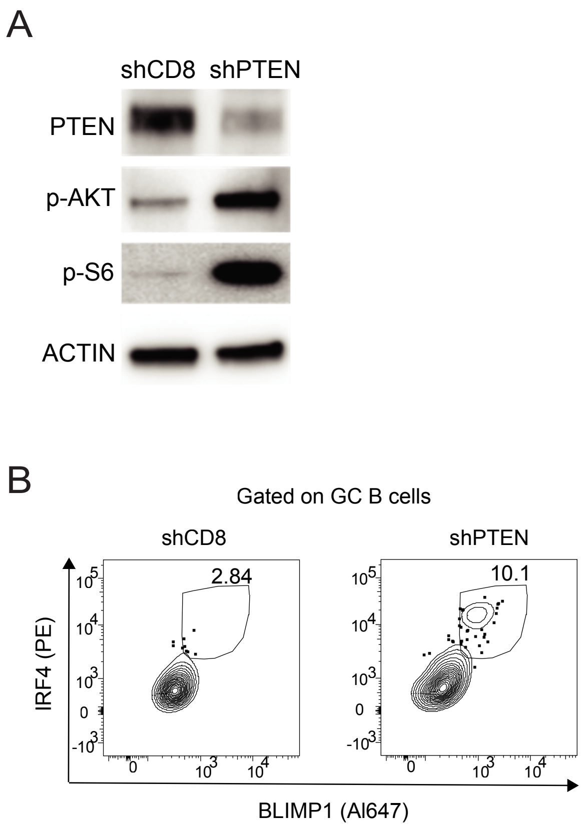

Fig. S2 | PTEN shRNA reduces PTEN expression and promotes GC B cell differentiation in vivo. (**A**) PTEN knockdown was validated in primary B cells transduced with a retrovirus expressing shRNA targeting CD8 (shCD8) or PTEN (shPTEN). Data shown is from a single experiment. (**B**) GC B cells transduced with shCD8 and shPTEN were co-transferred into recipient mice as outlined in Figure 2. Eight days post-transfer, the transduced GC B cells were analyzed for plasma cell (PC) precursor differentiation. Data represents one out of two recipient mice analyzed in a single experiment.
